# Impact of Cigarette Smoke Exposure on Innate Immunity: A *Caenorhabditis elegans* Model

**DOI:** 10.1371/journal.pone.0006860

**Published:** 2009-08-31

**Authors:** Rebecca M. Green, Fabienne Gally, Jonathon G. Keeney, Scott Alper, Bifeng Gao, Min Han, Richard J. Martin, Andrew R. Weinberger, Stephanie R. Case, Maisha N. Minor, Hong Wei Chu

**Affiliations:** 1 Department of Medicine, National Jewish Health, Denver, Colorado, United States of America; 2 Department of Molecular, Cellular and Developmental Biology, University of Colorado-Boulder, Boulder, Colorado, United States of America; 3 Department of Immunology, National Jewish Health, Denver, Colorado, United States of America; 4 Microarray Core, University of Colorado-Denver, Aurora, Colorado, United States of America; University of Pittsburgh, United States of America

## Abstract

**Background:**

Cigarette smoking is the major cause of chronic obstructive pulmonary disease (COPD) and lung cancer. Respiratory bacterial infections have been shown to be involved in the development of COPD along with impaired airway innate immunity.

**Methodology/Principal Findings:**

To address the *in vivo* impact of cigarette smoke (CS) exclusively on host innate defense mechanisms, we took advantage of *Caenorhabditis elegans* (*C. elegans*), which has an innate immune system but lacks adaptive immune function. *Pseudomonas aeruginosa* (PA) clearance from intestines of *C. elegans* was dampened by CS. Microarray analysis identified 6 candidate genes with a 2-fold or greater reduction after CS exposure, that have a human orthologue, and that may participate in innate immunity. To confirm a role of CS-down-regulated genes in the innate immune response to PA, RNA interference (RNAi) by feeding was carried out in *C. elegans* to inhibit the gene of interest, followed by PA infection to determine if the gene affected innate immunity. Inhibition of *lbp-7*, which encodes a lipid binding protein, resulted in increased levels of intestinal PA. Primary human bronchial epithelial cells were shown to express mRNA of human Fatty Acid Binding Protein 5 (FABP-5), the human orthologue of *lpb-7*. Interestingly, FABP-5 mRNA levels from human smokers with COPD were significantly lower (p = 0.036) than those from smokers without COPD. Furthermore, FABP-5 mRNA levels were up-regulated (7-fold) after bacterial (i.e., *Mycoplasma pneumoniae*) infection in primary human bronchial epithelial cell culture (air-liquid interface culture).

**Conclusions:**

Our results suggest that the *C. elegans* model offers a novel *in vivo* approach to specifically study innate immune deficiencies resulting from exposure to cigarette smoke, and that results from the nematode may provide insight into human airway epithelial cell biology and cigarette smoke exposure.

## Introduction

Human COPD patients show an impaired host innate immune response against airway bacterial infections [1], [2]. Innate immunity is the oldest host defense mechanism and is highly conserved across many species. In an attempt to look for an *in vivo* model, without the interference of the adaptive immune system, we decided to use the nematode *Caenorhabditits elegans (C. elegans)*. This organism lacks an adaptive immune system, but possesses a similar innate immune response to humans, including a toll-like receptor, several defensin-like proteins and other highly conserved innate immune mechanisms. *C. elegans* mounts an innate immune response against *Pseudomonas aeruginosa* (PA) – one of the known pathogens in COPD [3]. Additionally, *C. elegans* responds to nicotine, a major component of cigarette smoke, in a manner similar to that of mammals. Further, it converts nicotine to cotinine [4], showing that it breaks down nicotine in a similar manner to mammals and giving us a way to demonstrate that the animals are absorbing the smoke. Thus, *C. elegans* may be a good model to mimic human innate immune response to cigarette smoke exposure and bacterial infection. Finally, *C. elegans* has a short life span of approximately 14 days, allowing short duration smoke studies to cover a larger percentage of the life span. *C. elegans* is very well studied with all cells being fate-mapped. Its genome has been fully sequenced, and clones for RNA interference (RNAi) are available for most of the genes.

To discover novel innate immune genes regulated by cigarette smoke in humans, we used microarray and RNAi approaches to study cigarette smoke-exposed *C. elegans* with or without *PA* infection. We infected *C. elegans* with *Pseudomonas aeruginosa* strain PA14, a clinical isolate strain originally obtained from a human burn patient [5]. Non-infected animals were fed *E. coli* OP50, a non-pathogenic bacterial strain that is the standard laboratory food source used for *C. elegans* [6]. Using the above techniques, we successfully identified *lbp-7*, a lipid binding protein, which was down-regulated after CS exposure and played a role in innate immunity. Interestingly the human orthologue of this protein, FABP-5, was also present in human bronchial epithelial cells, was up-regulated in response to bacterial infection, and was down-regulated in COPD patients as compared to healthy smokers.

## Results

### *C. elegans* tolerated cigarette smoke (CS) exposure and converted nicotine from CS to cotinine

We exposed L4, late juvenile, *C. elegans* on agar plates with lids open to CS in a smoking chamber or, as a control, to filtered air for 1, 2, 3 or 4 hrs. We chose the L4 developmental stage so that nematodes were as close to fully developed as possible but were not yet fertile and egg-laden, as nicotine has been shown to affect egg laying behavior [7]. After 24 or 48 hrs, when *C. elegans* had developed into adults, nematode survival was assessed. CS exposure of up to 4 hrs did not affect the survival of *C. elegans* after 24 hrs of CS withdrawal (n = 300 worms per each of 1, 2 and 3 hrs of CS exposure). At 48 hr post-CS, a few of the nematodes exposed to CS for 4 hrs died, but there was no statistically significant difference (98%±0.5% survival for CS vs. 100% survival for air, n = 300, p = 0.28). Exposure to CS for more than 3 hrs also caused some desiccation of the plates.

**Figure 1:**
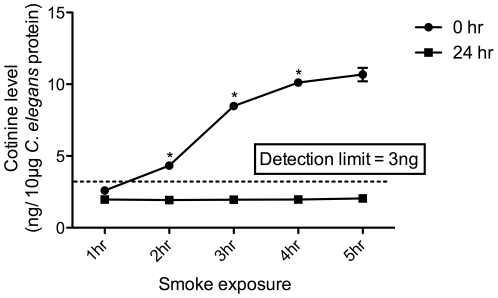
Cotinine levels increase as a function of the length of exposure to cigarette smoke (CS). Cotinine levels in *C. elegans* show dose dependent increases based on the amount of time exposed to CS. 2 replicates are shown. An * represents a data point with a statistically significant difference from either of its neighbors. Any data below 3 ng cotinine per 10 µg of total protein fell below the detectable level. Animals were harvested immediately following (0 hr) or 24 hrs following CS exposure.

In order to prove that *C. elegans* were able to absorb chemicals from the CS exposure, levels of cotinine, a nicotine metabolite, were measured immediately following (0 hr), 24 hrs post, and 48 hrs post CS. We observed a dose-dependent increase in cotinine at 0 hr. By 24 hours, the animals have metabolized the cotinine, and levels have fallen back below detectable levels (Figure 1). Cotinine was also undetectable 48 hrs after CS exposure (data not shown).

### CS exposure impaired intestinal bacterial clearance

**Figure 2:**
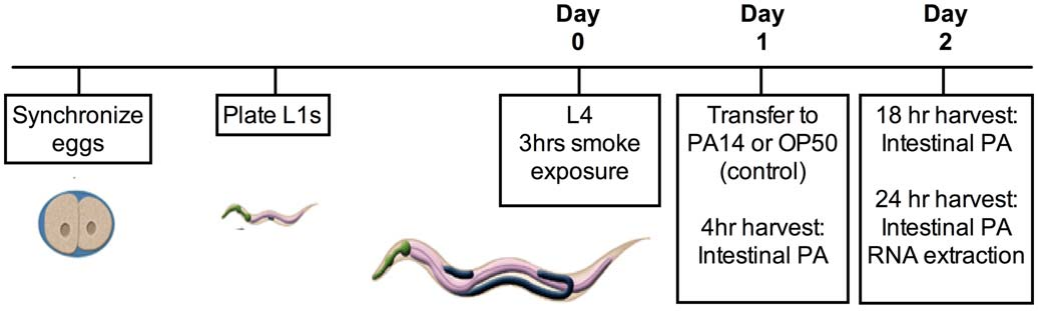
Cigarette smoke exposure and infection timeline. L1 nematodes were plated on NGM agar and allowed to mature to the L4 stage. At this point animals were exposed to cigarette smoke for 3 hrs and allowed to mature on the same plates, day 0. After 24 hrs, the animals were transferred to infection or control plates, day 1. Animals were harvested at 4, 18, or 24 hrs post infection for intestinal PA quantification and RNA extraction.

**Figure 3:**
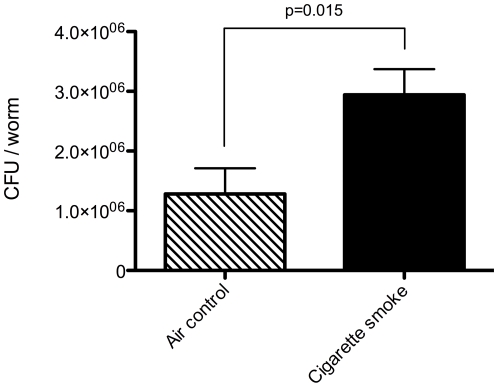
Cigarette smoke exposure impaired intestinal bacterial clearance. Mean *Pseudomonas aeruginosa* (PA) loads in the intestines of *C. elegans* exposed to air control or cigarette smoke for 3 hrs. Colony forming units (CFU) were determined 24 hrs post PA infection. Data represents 3 independent trials.

A timeline of the experimental design for CS exposure and infection of *C. elegans* is shown in Figure 2. *C. elegans* were exposed to CS for 3 hrs (the longest time that did not cause desiccation of the agar plates), and were subsequently allowed to grow for an additional 24 hrs on the same plates that had been CS-exposed. We monitored *Pseudomonas aeruginosa* (PA) load in the intestines at 4 and 18 hrs post-infection and did not observe a significant change in PA load (data not shown). However, by 24 hrs after the start of the infection, *C. elegans* showed increased levels of PA in the intestines (Figure 3). The 24 hr time point was therefore used for microarray analysis to identify genes that could be involved in impaired bacterial clearance (see below).

To confirm that the increase in intestinal PA load following CS exposure was due to infection and not simply due to the animals eating more, we analyzed pharyngeal pumping rates 24 and 48 hrs post CS exposure, the period when *C. elegans* would have been infected with PA. As the nematode eats, it is easy to observe the pumping pharynx, and the rate of pumping has been shown to correlate to the amount of food consumed [8]. No differences in pumping rates were found at 48 hr post-CS exposure between CS-exposed and control animals on either OP50 or PA14 plates (CS+OP50, 210±6 pumps/min vs. air+OP50, 216±6 pumps/min, p = 0.58 and CS+PA, 184±4 pumps/min vs. Air+PA, 186±4 pumps/min, p = 0.87) (15 animals scored on each of three plates per condition). However, at 24 hr post-CS exposure, immediately before the nematodes would be exposed to PA, there was a slight (about 10%), but statistically significant decrease in pharyngeal pumping rates (CS, 204±4.8 pumps/min vs. control, 228±2.4 pumps/min, p = 0.002, 15 animals scored on each of three plates per condition). Thus, pharyngeal pumping rates following CS exposure are unchanged or slightly **decreased**. A decrease in pumping would be expected to cause a decrease in intestinal bacteria, as less bacteria are likely entering the nematode. This decrease, therefore, would not account for the approximately 100% **increase** of intestinal PA levels in CS-exposed over non-CS-exposed *C. elegans*, suggesting that CS is affecting PA infection and not merely nematode feeding.

### Effects of CS on gene expression in *C. elegans*

We exposed *C. elegans* to either CS or PA and monitored changes in gene expression using microarrays as described in the Materials and Methods. Significant changes in genes expression were identified using the MAS5 algorithm [9]. We identified 117 genes whose expression was down-regulated ≥2-fold by CS exposure (Table S1), while only 19 genes were up-regulated by CS≥2-fold (Table S2). In contrast to CS exposure, exposure to PA led to the up-regulation of 156 genes ≥2-fold (Table S3) and the down-regulation of only 65 genes (Table S4). Of the 156 genes up-regulated by PA, 5 have been reported previously to be involved in the *C. elegans* response to PA during a shorter (4 or 8 hrs) infection [10]. Table 1 shows these five genes in **bold**.

**Table 1:** Fold changes of *C. elegans* genes – microarray analysis.

Worm base ID	Alternate ID	Description and protein ID	Smoke vs. Air	PA14 vs. OP50	Human Orthologue
**T22G5.2**	*lbp-7*	Fatty-acid binding protein (CE13984)	−3.03	+3.03	FABP-5, 5L2, 5L7, 7, 9
D2045.8		TNF-alpha induced Protein B12 (CE00608)	No Change	+3.24	TNFAIP1
F44E5.4	*hsp-70*	Heat shock hsp70 proteins (CE18679)	No Change	+3.03	HSP-70
F13B10.1	*tir-1*	TIR (Toll/Interleukin 1 Receptor) domain protein (CE15818)	−3.24	+3.48	Myd88 family, SARM
**M60.2**		M60.2 (CE04775) new id (CE28630)	−6.49	+4.92	Precursor to human placental protein 11
**F35E8.8**	*gst-38*	Glutathione S-transferases (CE15958)	No Change	+59.71	PGDS
**C54D1.2**	*clec-86*	C-type lectin (CE06978)	No Change	+12.99	REG1A
F46B6.8		Lipase (CE05874)	No Change	+4.28	LIPF
Y46H3A.3	*hsp-16.2*	Heat shock protein (CE22002)	2.14	+3.03	CRYAB
T27E4.8	*hsp-16.1*	Heat shock protein HSP16-1 (CE14249)	No Change	+2.63	CRYAB
F26E4.12		Glutathione peroxidase (CE09696)	No Change	+2.46	GPX4
**T23H4.3**	*nas-5*	zinc metalloprotease (CE25126)	No Change	+2.29	MEP1B
F58F9.7		acyl-coenzyme A oxidase (CE07304)	No Change	+2.14	ACOX3
Y54G11A.13	*ctl-3*	Catalase (CE22478)	No Change	+2.00	CAT
F18E2.1		Acid phophatase like (CE05662)	No Change	−2.29	AC011443.6
F32H2.6	*phi-46*	Fatty acid synthase (N-terminus) (CE09881)	No change	−2.46	FASN
F28A10.6	*acdh-9*	Acyl-coA dehydrogenase (CE19411)	−3.48	−2.46	ACAD8
C11E4.1		Glutathione peroxidase (CE08101)	−2.46	−2.63	GPX5
F18E3.7		D-amino acid oxidase (CE07083)	−3.24	−3.24	DAO/DAMOX
F44G3.2		Arginine kinase (CE16034)	No Change	−34.29	CKB

Animals were harvested 48 hrs after cigarette smoke or air (control), and 24 hrs after *Pseudomonas aeruginosa* (PA14) or sham infection. Genes presented above the double line are the genes selected for RNAi knockdown. Genes shown are either genes commonly thought to be involved in immune responses or genes that showed changes in at least 2 categories. Bolded genes were previously reported to play a role in host immune response [5]. “+” or “−” indicate an up-regulation or a down-regulation of the gene, respectively.

To select genes for further study, we selected only genes with human orthologues. Of these, we then selected genes that showed a change in response to infection and either exhibited a change in response to CS, or that had been previously hypothesized to be involved in human COPD or innate immunity. We pared these genes to 20 final candidate genes ( Table 1 ). From this list of 20 genes, we selected 5 genes to investigate further in RNAi-mediated gene inhibition studies (five genes listed above the double line in Table 1). These final five candidate genes were selected based on availability of the RNAi clones for the genes that showed at least a 3-fold change from both PA and CS as well as *hsp-70* and the TNFα induced protein based on previous publication [11], [12].

### *lbp-7* RNAi treatment increased intestinal bacterial load

By feeding the nematodes a strain of *E. coli* expressing dsRNA of a single gene (RNAi by feeding [13]), we were able to inhibit each of these five candidate genes to test them for a role in host defense. The five available clones listed above the double line in Table 1 are: lipid binding protein 7 [*lbp-7*, (worm base ID: T22G5.2)], M60.2, D2405.8 (which is homologous to a human TNF-α induced protein), F44E5.4 (an hsp70 like protein), and *tir-1*(a homolog of mammalian SARM and regulator of *C. elegans* innate immunity [14]).

**Figure 4:**
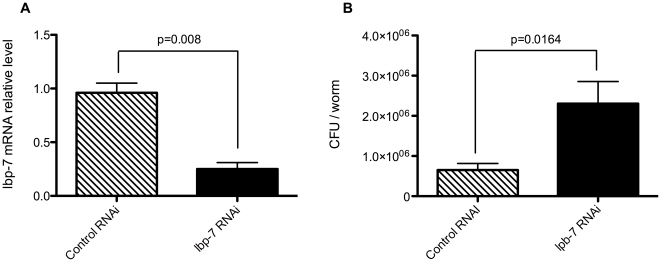
RNAi-mediated inhibition of *lbp-7* increases intestinal bacteria. A: RNAi against *lbp-7* down-regulates its expression approximately 3.8 fold compared to control (L4440) vector. B: RNAi by feeding of *lpb-7* in *C. elegans* results in statistically significant increases in intestinal PA14 loads compared to the control vector.

RNAi-treated animals were then infected with PA for 24 hrs. Inhibition of *lbp-7* significantly increased intestinal PA14 load, when compared to a control RNAi treatment (Figure 4B). RNAi-induced reduction of *lpb-7* gene expression was confirmed by real-time quantitative PCR (qRT-PCR) (Figure 4A). Specificity of the *lbp-7* RNAi was further confirmed by qRT-PCR for *lbp-8*, a closely related family member of *lbp-7*. No significant differences of *lbp-8* mRNA levels were seen between the *lbp-7* knockdown and the control (*lbp-8* mRNA levels: 0.073±0.012 vs. 0.052±0.012, n = 3, p = 0.257). RNAi of the other 4 genes did not result in a significant change in intestinal PA load as compared to the control RNAi treatment (data not shown).

As further confirmation of the role of *lbp-7* in innate immunity, we monitored *lbp-7* expression when *C. elegans* was exposed to PA. PA infection increases *lbp-7* mRNA approximately 4 fold (*lbp-7* mRNA levels: 0.329±0.204 [(−)] vs. 1.321±0.205 [PA], n = 5 p = 0.0014), giving more evidence for *lbp-7*'s role in the innate immune response.

### CS attenuates *lbp-7* induction during *Pseudomonas aeruginosa* (PA) infection

*lbp-7's* role in host defense and CS response is further supported by our microarray data, which shows a 2.5 fold reduction of expression of *lbp-7* in CS+PA treated animals compared to PA alone. This microarray result was confirmed by qRT-PCR, which showed approximately a 50% reduction in *lbp-7* mRNA levels by CS when comparing CS+PA treatment to PA treatment alone (*lbp-7* mRNA levels: 1.321±0.205 [(−)+PA] vs. 0.679±0.193 [CS+PA], n = 5, p = 0.028).

### Human primary airway epithelial cells expressed FABP-5, the human orthologue of *lbp-7*

**Figure 5:**
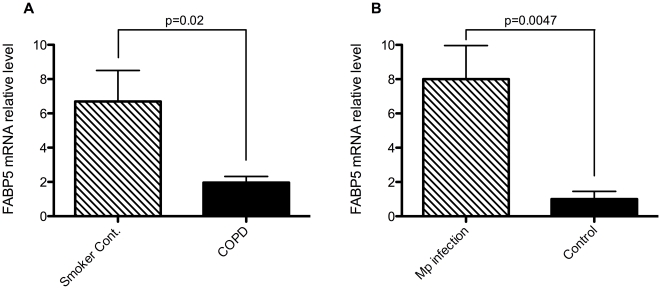
FABP-5 expression in primary human bronchial epithelial cells. A: FABP-5 mRNA is down-regulated approximately 3.4 fold in un-cultured primary human brushed bronchial epithelial cells from COPD patients compared to smoker controls. B: *Mycoplasma pneumoniae* (Mp) infection up-regulates FABP-5 mRNA approximately 7 fold in cultured human bronchial epithelial cells under air-liquid interface conditions. Cells were infected for 7 days to mimic chronic infections seen in COPD patients.

To examine the relevance of genes discovered using the *C. elegans* model to human airway cell biology, we examined expression of FABP-5 (UniProtKB/Swiss-Prot: FABP5_HUMAN, Ensembl transcript ID: ENST00000297258, Ensembl protein id: ENSP00000297258), a human orthologue of *lbp-7*, in primary human bronchial epithelial cells obtained through bronchial brushings from smokers with (n = 5) or without (n = 4) COPD. The purity of epithelial cells was greater than 95% based on cell morphology and staining with cytokeratin peptide 18 on cell cytospin preparations (data not shown). First, FABP-5 mRNA was detected in epithelial cells from both groups of human subjects. Second, the mRNA levels of FABP-5 were significantly lower (about 70%) in smokers with COPD than smokers without COPD (Figure 5A).

*lbp-7* expression was up-regulated by PA infection in *C. elegans* (see above). We therefore monitored FABP-5 expression in cultured human bronchial epithelial cells to see if the human orthologue was similarly regulated by pathogen. Since PA in cultured cells would lead to cell death within several hours of infection [15], [16], we infected cultured human bronchial epithelial cells under the air-liquid interface culture (ALI) with *Mycoplasma pneumoniae* (Mp). Mp has been shown to be involved in COPD and can infect cells for long periods of time (up to 14 days) without causing cell death, reflecting the chronic nature of the infection in humans [17]. Cell culture data from cells of various patient types (COPD, smoker and non-smoker controls) were merged following independent analysis showing similar trends. Previously, we have shown that after weeks in culture cells tend to lose their phenotypic distinctions [13]. Using real-time quantitative PCR we found that FABP-5 expression was up-regulated 7 fold in response to Mp infection (Figure 5B). FABP5, therefore, does appear to be involved in the human airway innate immune response.

## Discussion

Our goal for this study was to determine if the nematode *Caenorhabditits elegans* could be used as a model of the innate immune response found in humans exposed to cigarette smoke. First, we show that *C. elegans* can absorb nicotine from cigarette smoke and break it down similarly to humans. Second, we demonstrated that exposure to CS modulated gene expression and impaired bacterial clearance in the *C. elegans*' intestine. Third, we found that CS-induced down-regulation of *lbp-7* and *lbp-7* was up-regulated in response to bacterial infection in wild-type *C. elegans*. Further, inhibiting *lbp-7* expression via RNAi increases intestinal bacteria. Together, these data suggest that *lbp-7* is in part responsible for the impaired intestinal bacterial clearance seen in the CS-exposed nematodes. Lastly, we showed that expression of FABP-5, the human orthologue of *lbp-7*, was also up-regulated by bacterial infection. Moreover, FABP-5 expression was down-regulated in bronchial epithelial brushings from human COPD patients relative to healthy smokers, suggesting that FABP-5 could play a role in human disease.

While many previous studies have used *C. elegans* to study the innate immune response [18], [19], we are the first group to expose *C. elegans* to CS. Human epithelial cell culture has limitations, primarily those of any *in vitro* model [20]. *C. elegans*, as an *in vivo* system, can be subjected to whole cigarette smoke exposure, and may help overcome some of the weaknesses in human epithelial cultures. *C. elegans* down-regulated many genes in response to CS including several host defense genes. This down-regulation may be responsible for CS-induced impaired intestinal clearance of *Pseudomonas aeruginosa* PA14, a bacterium involved in both human and nematode disease. Our microarray studies examining the effect of *Pseudomonas aeruginosa* PA14 on *C. elegans* at 24 hrs post-infection revealed changes in expression of many genes including genes reported to be regulated by PA at earlier time points. We chose several genes regulated by PA exposure for further analysis.

To confirm that the changes in bacterial load caused by CS were not simply caused by the animals eating more, we compared pharyngeal pumping rates between *C. elegans* with or without CS exposure. We found either a slight decrease or no statistical difference, depending on time point. A decrease in pumping rates would suggest that the nematodes are eating less, so it is even more striking to see the increase in intestinal PA. In order to better understand the mechanism behind the impaired bacterial clearance seen in CS-exposed animals, we used RNAi by feeding to knock down expression of five candidate genes reported in the results. Of these candidate genes, *lbp-7* knockdown resulted in an increased bacterial load. *lbp-7* encodes a predicted intracellular fatty acid binding protein that is anticipated to function as an intracellular transporter for small hydrophobic molecules such as lipids and steroid hormones [21]. Large-scale *C. elegans* RNAi screens have not shown obvious phenotypic abnormalities when *lbp-7* is inhibited. However, the precise role of *lbp-7* in *C. elegans* development and/or behavior is not yet known. *lbp-7* is expressed in the *C. elegans* intestine. We are the first to demonstrate that this gene is important in the immune response to bacterial infection. Since the expression of *lbp-7* is induced less strongly in the presence of CS and infection compared to infection alone, it is likely involved in the impaired bacterial clearance seen in smoke-exposed animals.

In future studies we would like to further dissect the interplay between *lbp-7* and CS. For example, overexpressing *lbp-7* would be useful to define the contribution of this gene in PA infection in the presence or absence of smoke exposure.

The fatty acid binding protein FABP-5, or E-FABP (epidermal fatty acid binding protein), a human orthologue of *lbp-7*, has been shown to be present in human lung endothelium and airway secretory cells such as Clara cells and goblet cells [22]. It is a 15 kD cytoplasmic protein that specifically binds fatty acids [23]. We show that FABP-5 is up-regulated in airway epithelial culture upon bacterial infection, demonstrating its potential role in the innate immune response. More importantly, FABP-5 is down-regulated in COPD patients compared with smoker controls, implying its involvement in COPD pathogenesis. Further studies such as knockdown or overexpression experiments in human airway epithelial cells should be completed to help us further understand FABP-5's roles in the human innate immune response in COPD and other chronic lung diseases presenting persistent airway bacterial infections.

In summary, our current study offers a novel model to exclusively investigate the role of innate immunity in host defense in the context of cigarette smoke exposure. This model should improve our understanding about the contribution of innate immunity to the nature of bacterial infections in patients with debilitating chronic lung diseases including COPD.

## Materials and Methods

The human research protocols listed in this paper were approved, via written approval, by the National Jewish Health Institutional Review Board (IRB), and all patients gave written informed consent (protocol number: HS2134). Other work was not subject to IRB regulation.

### Cigarette smoke exposure in *C. elegans*

*C. elegans* eggs were isolated and synchronized using an alkaline hypochlorite solution and incubated overnight at room temperature with shaking, as described previously [24]. L1s were plated on nematode growth medium (NGM) agar plated with *E. coli* OP50 and allowed to grow at 20°C until late L4. A Teague-10 cigarette smoke exposure system was used to expose *C. elegans* to whole cigarette smoke. In order to control for any effect of the bacterial lawn thickness on smoke exposure, all plates were plated with 500 µl of OP50 grown overnight to an OD_595_ of 0.5. *C. elegans* were placed in the smoke chamber for 1, 2, 3 or 4 hrs at 22–25°C and exposed to a mixture of mainstream (11%) and sidestream (89%) cigarette smoke with a carbon monoxide (CO) concentration of 190 to 300 ppm and a total suspended particle (TSP) of 85 to 120 mg/m^3^ [25]. The nicotine concentration was 6 mg/m^3^. Control animals were exposed to 3 hrs of filtered air. All *C. elegans* were allowed to finish maturing for 24 hrs on the same (smoke or air control) plates.

### Cotinine analysis

Approximately 500 animals were collected from each condition at 0, 24 or 48 hr following smoke exposure (n = 3), washed 3x to remove any bacteria, then placed in a cell lysis buffer (20 mM Tris·Cl, 500 mM NaCl, 0.05% (v/v) Tween 20, 0.2% (v/v) Triton X-100, pH 7.5) and frozen at −80°C until needed. Frozen pellets were then homogenized on ice for 30 seconds using a motorized pestle. then for 15 min in a sonicator. Total protein was analyzing using the Pierce Scientific BCA kit following the manufacturer's instruction. 10 µg of protein was then analyzed with the Calbiotech Cotinine Elisa (Cat#CO096D) following the manufacturer's protocol.

### Pseudomonas aeruginosa (PA14) infection

PA14, a gift from Dr. Quinn Parks, was grown in LB broth overnight to an OD_595_ of 0.5. 500 µl of culture was plated on each 60 mm dish of NGM media, spread evenly and allowed to dry overnight at room temperature. About 60 nematodes, 24 hrs post-cigarette smoke or air control exposure, were transferred to each plate.

### Microarray analysis of *C. elegans* genes expression

To determine the effect of CS on gene expression, CS or air (control) animals were collected 48 hrs post exposure for RNA harvest. For the PA infection studies, animals were collected 24 hrs post infection. Both groups were age matched at the time of harvest. RNA was isolated as described below.

The total RNA was subjected to the standard microarray procedure [26], [27] to analyze gene expression by using the *C. elegans* whole genome GeneChip (Affymetrix, Palo Alto, CA) that contains over 22,500 transcripts of confirmed *C. elegans* cDNA. Sequences Arrays were read at a resolution of 2.5 to 3 microns using the GeneChip Scanner 3000 (Affymetrix).

Gene expression data were analyzed with Affymetrix GeneChip Operating Software using the MAS5 algorithm. Fold changes in RNA expression were calculated by comparing the CS exposure or PA14 infection sample to the corresponding control sample. Genes were defined as differentially transcribed if they were induced or repressed at least two-fold. All microarray data reported in the manuscript is described in accordance with MIAME guidelines.

### RNA analysis

Total RNA was isolated, using the TRIzol reagent (Invitrogen), from approximately 100 adult nematodes that were previously rinsed in M9 buffer to remove surface bacteria, following the manufacturer's instructions with the following modification. Briefly, nematodes were frozen in 250 µl of TRIzol, and then homogenized while defrosting using a motorized pestle. RNA was cleaned using the Ambion DNAse kit, followed by reverse transcription using the Applied Biosystems kit.

### Quantification of intestinal PA loads

Quantification of intestinal PA loads was performed as described by Moy et al [28] in at least three independent experiments. Briefly, ten nematodes were removed from each plate and washed three times with 250 µl of M9 salts containing 1 mM sodium azide in a 1.5 ml microcentrifuge tube. 50 µl of the solution was then collected for determination of external bacterial load. 400 mg of 1.0 mm silicon carbide beads were added to the remaining solution to homogenize the nematodes by vortexing for 1 min. The homogenized nematode solution and the external wash solutions were serially diluted and plated onto cetramide agar selective media for counting colony-forming unit (cfu). Pharyngeal counting was used to confirm that the animals were consuming the food source at the same rate. 15 animals from each of three plates were analyzed for each condition, and the number of pharyngeal pumps was counted for 30 seconds and then multiplied by two to reflect pumps/min.

### RNA interference (RNAi) by feeding in *C. elegans*

RNAi by feeding was conducted as described by Kamath et al [29]. RNAi-transfected bacterial colonies were obtained from Geneservice Ltd, UK. The empty L4440 vector was used as a control as described in our previous publication [30]. Briefly, colonies were picked from LB plates containing 12.5 µg/mL tetracycline and 25 µg/ml carbenecillin that had been allowed to grow overnight at 37°C. The desired colony was transferred into 200 ml LB broth containing 25 µg/ml carbenecillin and grown overnight at 37°C with shaking, which yielded an OD_595_ of 0.4 or higher. 500 µl of culture was inoculated onto NGM containing 1 mM IPTG and 25 µg/ml carbenicillin. L4 nematodes were transferred to the RNAi plates and allowed to mature for 48 hrs before PA infection.

### Real-time quantitative PCR

Applied Biosystems gene expression assays were used for real-time PCR against *C. elegans gdp-1* (endogenous control) (Assay ID: Ce02617309_gH), *lpb-7* (Assay ID: Ce02484404_g1), *lbp-8* (Assay ID: Ce02484416_s1), human FABP-5 (Assay ID: Hs02339437_g1) and human GAPDH (endogenous control) (Cat#4352934E). Data were analyzed using the comparative cycle of threshold method [17].

### FABP-5 gene expression analysis in primary human airway epithelial cells

The human research protocol (protocol number: HS2134) was approved by the institutional review board (IRB). The protocol of bronchoscopy and bronchial brushings was also approved by the IRB, and all research subjects gave written informed consent. To test if a *C. elegans* gene orthologue was altered in human smokers with COPD, we examined the mRNA levels of a gene of interest (e.g., FABP-5) in brushed bronchial epithelial cells using real-time quantitative PCR. Bronchial brushings were performed in healthy smokers (n = 4) and smokers with COPD (n = 5), as previously described, with the modification of using a sterile single-sheathed nylon cytology brush specifically designed for bronchial brushing (Medical Engineering Reorder #CF-001, Durham, NC) [31]. COPD patients had Global Initiative for COPD (GOLD) stages between II and IV. The clinical features of the healthy smokers and COPD patients are presented in Table 2. A total of four to six bronchial brushings from the right or left lower lung lobes were obtained. Cells (1-2×10^6^, >95% being epithelial cells as confirmed by immunostaining of cytokeratin peptide 18) were placed into 10 ml of ice-cold phosphate-buffered saline (PBS), centrifuged, washed in ice-cold F12 medium, and resuspended in 1 ml of serum-free hormonally supplemented bronchial epithelial growth medium (Clonetics, San Diego, CA) containing 50 µg/ml of gentamicin and 50 µg/ml of amphotericin. About 0.5×10^6^ cells were processed for RNA extraction, followed by reverse transcription and real-time quantitative PCR.

**Table 2:** Characteristics of Study Participants.

	GOLD COPD Stages	Gender	Age	Smoking (pack-years)	FEV_1_% predicted	FVC% predicted	FEV1/ FVC%
Control	N/A	f	52	45	108	99	86
Control	N/A	m	55	43	79	74	81
Control	N/A	m	44	30	122	121	82
Control	N/A	m	50	30	104	94	86
Mean±SEM			50.2±2.3	37±4.1	103.2±8.9	97±9.6	83.8±1.3
COPD	3	f	67	44	33	85	30
COPD	2	m	71	112	34	34	75
COPD	4	m	68	60	29	58	34
COPD	4	m	68	45	23	47	36
COPD	4	f	66	55.5	15	34	34
Mean±SEM	3.4±0.4		68.0±0.8	63.3±12.5	26.8±3.5	51.6±9.4	41.8±8.3

GOLD = Global Initiative for Chronic Obstructive Lung Disease. FEV1 = Forced expiratory volume in the first second, FVC = Forced vital capacity.

### Primary bronchial epithelial cell air-liquid interface culture

Brushed bronchial epithelial cells were seeded onto 60 mm collagen-coated tissue culture dishes at 1×10^5^ cells and incubated at 37°C with 5% CO_2_. At 80% confluence, they were trypsinized and seeded on collagen-coated transwell inserts in 12-well plates [31]. Cells remained immersed in medium for seven days to reach confluence, then were shifted to air-liquid interface (ALI). After 10 days of ALI culture, cells demonstrated mucociliary differentiation, and were infected with *Mycoplasma pneumoniae* (Mp, 10 cfu/cell) at the apical surface. Seven days post-Mp infection, cells were harvested into TRIzol (Invitrogen) for total RNA extraction.

### Mp Preparation and Culture

Mp was prepared for cell culture as previously described [17]. To quantify Mp in apical supernatants of epithelial cells, PPLO plates with apical supernatant were incubated at 37°C with 5% CO_2_ for seven days for CFU counting.

### Statistical Analysis

Data are presented as means±SEM and were compared using a 1-way ANOVA test for comparisons of 3 groups or more and a Student *t*-test for 2 group comparisons. A value of p<0.05 was considered significant. All values represent at least 3 independent trials.

## Supporting Information

Table S1 Microarray Smoke vs. Air - Reduced Genes. This is the raw microarray data showing all genes with a 2-fold or greater reduction between smoke exposure and air controls.
(0.12 MB DOC)

Table S2 Microarray Smoke vs. Air - Increased Genes. This is the raw microarray data showing all genes with a 2-fold or greater increase between smoke exposure and air controls.
(0.04 MB DOC)

Table S3 Microarray Pseudomonas vs. Control - Decreased Genes. This is the raw microarray data showing all genes with a 2-fold or greater reduction between Psuedomonas exposure and air controls.
(0.18 MB DOC)

Table S4 Microarray Pseudomonas vs. Control - Increased Genes. This is the raw microarray data showing all genes with a 2-fold or greater increase between Psuedomonas exposure and air controls.
(0.09 MB DOC)
