# Supplementary material for: Impact of Cigarette Smoke Exposure on Innate Immunity: A Caenorhabditis elegans Model
Source: PLoS One. 2009 Aug 31;4(8):e6860. doi: 10.1371/journal.pone.0006860 (PMC2729919; doi:10.1371/journal.pone.0006860)
Supplement: Table S1 — Microarray Smoke vs. Air - Reduced Genes. This is the raw microarray data showing all genes with a 2-fold or greater reduction between smoke exposure and air controls. (0.12 MB DOC) [file pone.0006860.s001.doc]

**Table S1.** *C. Elegans* genes (n = 117) reduced by cigarette smoke exposure (Smoke vs. Air)

| **Worm base ID** | **Fold reduction** | **Description and protein ID** |
| --- | --- | --- |
| F38A3.1 | 39.4 | Collagen (CE02213) |
| F41F3.4 | 21.1 | Cuticle collagen (CE07218) |
| C39H7.1 | 19.7 | Casein kinase (CE06942) |
| F23B2.2 | 16.0 | PDZ domain (CE09582) |
| C10G11.8 | 13.9 | ATPase (CE27676) |
| C17C3.12 | 12.1 | Acyl-CoA dehydrogenase (CE03983) |
| F38E11.1 | 12.1 | Alpha-B-crystallin (CE03293) |
| ZK1248.6 | 12.1 | Major sperm protein (CE02806) |
| F42D1.2 | 12.1 | Tyrosine aminotransferase (CE10298) |
| T03F1.5 | 12.1 | Serine-threonine protein phosphatase PP1 (CE13104) |
| C10C5.3 | 11.3 | Aminoacylase-1 (CE03011) |
| F08F3.7 | 10.6 | Cytochrome P450 (CE09262) |
| ZK938.1 | 10.6 | Serine-threonine protein phosphatase (CE15483) |
| ZK1251.1 | 8.6 | Histone H2A (CE03848) |
| F10D2.9 | 7.5 | Stearoyl-CoA desaturase (CE09320) |
| F26A3.4 | 6.5 | Protein-tyrosine phosphatase (CE09669) |
| T05A10.3 | 6.5 | Transthyretin-like family (CE03635) |
| M60.2 | 6.5 | Similarity to Pfam domain PF09412 (CE04775) |
| C10C5.4 | 6.1 | Aminoacylase-1 (CE20491) |
| F25H5.1B | 5.7 | LIM domain containing proteins (5 domains) (CE15896) |
| F28F8.2 | 5.7 | Long-chain-fatty-acid-CoA ligase (CE09756) |
| K08E3.1 | 5.3 | Tyrosinase (CE18864) |
| F37B4.7 | 5.3 | Folate transporter (CE17796) |
| F22E10.5 | 5.3 | Phosphotransferase (CE05695) |
| K08C7.2 | 5.3 | Dimethylaniline monooxygenase (CE21038) |
| F35G2.4 | 5.3 | Prolyl 4-hydroxylase alpha subunit (CE05811) |
| ZC101.2A | 5.3 | UNC-52 membrane proteoglycan (CE15028) |
| Y38H8A.3 | 5.3 | Serine-threonine kinase (CE18365) |
| F45E4.1 | 5.3 | ADP-ribosylation factor (CE10492) |
| M02F4.8 | 4.9 | MIP family protein (CE04769) |
| C31C9.1 | 4.9 | Apical gut membrane protein (CE08496) |
| F47B10.7 | 4.9 | Acyl-CoA-binding protein (CE03357) |
| F53F1.4 | 4.9 | Cuticlin (CE10938) |
| K08E5.2 | 4.9 | Yeast ORF YCR37C (CE01602) |
| W02B12.12 | 4.6 | Serine-threonine kinase (CE21239) |
| F58A3.5 | 4.6 | Transthyretin-like family (CE11360) |
| ZK909.4 | 4.6 | Albumin D-binding protein like (CE15479) |
| F36H12.7 | 4.6 | Major sperm protein MSP-19 (CE02806) |
| K02D7.1 | 4.6 | Phosphorylase (CE17994) |
| C23G10.4A | 4.6 | tRNA processing protein SEN3 (CE04049) |
| M176.6 | 4.6 | Tyrosine-protein kinase (KIN15-KIN16 subfamily) (CE12470) |
| F55F3.3 | 4.6 | Sodium and potassium ATPases (CE18746) |
| F47B10.2 | 4.6 | Histidine ammonia-lyase (CE03352) |
| F32A5.2 | 4.3 | Peroxidase (CE01934) |
| g9966516 | 4.3 | Tropomyosin isoform IV (AF298180.1) |
| F41E7.5 | 4.3 | Collagen-like repeats (CE03305) |
| F46G10.6 | 4.3 | Helix-loop-helix DNA-binding domain (CE05882) |
| R04B5.9 | 4.3 | UDP-glucuronosyltransferase (CE06219) |
| F15B9.8 | 4.3 | Thrombospondin type 1 domain (CE20686) |
| C49F5.1 | 4.3 | Adenosylmethionine synthetase (CE08852) |
| T28F3.4 | 4.3 | Sugar (and other) transporters (CE09680) |
| C02C2.1 | 4.3 | Tyrosinase (CE06754) |
| K12G11.3 | 4.3 | Alcohol dehydrogenase (CE12212) |
| C09G4.2 | 4.3 | cAMP-dependant protein kinase (CE03979) |
| F09E10.3 | 4.0 | Short-chain alcohol dehydrogenase (CE04339) |
| F01G12.5A | 4.0 | alpha-2 type IV collagen (CE04334) |
| K02B9.4 | 4.0 | GATA-binding transcription factor like (CE23844) |
| F32B5.1 | 4.0 | Phosphotransferase (CE28230) |
| C17G1.7 | 4.0 | Cysteine synthase (CE08286) |
| H01A20.1 | 4.0 | Zinc finger, C4 type (two domains) (CE18798) |
| F20D1.5 | 4.0 | ADP-ribosylation factor (CE09498) |
| F58A4.7B | 4.0 | Helix-loop-helix DNA-binding domain (CE20890) |
| M03B6.2 | 3.7 | Monocarboxylate transporter like (CE12336) |
| ZK822.5 | 3.7 | Sodium-proline symporter like (CE18466) |
| T22C8.2 | 3.7 | Hyaluronidase (CE02350) |
| F07H5.9 | 3.7 | Acid phosphatase (CE03158) |
| T09A5.1 | 3.7 | Calcium binding protein (CE03645) |
| C01G6.1A | 3.7 | Glycerol uptake facilitator protein (CE00863) |
| AU109732 | 3.7 | Galactosyltransferase (CE03878) |
| T22B2.4 | 3.7 | RNA-binding protein (CE13934) |
| C14F11.6 | 3.7 | DTDP-4-dehydrorhamnose 3,5-epimerase (CE02482) |
| ZK892.1 | 3.7 | Lec-3 galactoside-binding lectin (CE24743) |
| M6.1 | 3.7 | Intermediate filament protein A (CE07382) |
| K02G10.7 | 3.5 | Transmembrane channel protein (CE04707) |
| M163.3 | 3.5 | Histone H1(CE12450) |
| AC3.8 | 3.5 | UDP-glucuronosyltransferase (CE05138) |
| K10B2.2 | 3.5 | Carboxypeptidase (CE02009) |
| F28A10.6 | 3.5 | Acyl-coA dehydrogenase (CE15958) |
| F14E5.4 | 3.2 | Acid phosphatase (CE03207) |
| F13B10.1 | 3.2 | Toll/Interleukin 1 Receptor Domain protein (CE15818) |
| T03F7.7 | 3.2 | Candida SEC14 cytosolic factor like (CE20032) |
| C04F6.3 | 3.2 | Glycosyl hydrolase (family 18) (CE03923) |
| F40F9.9 | 3.2 | MIP transmembrane protein (CE20771) |
| F18E3.7 | 3.2 | D-amino acid oxidase (CE07083) |
| T01C8.5 | 3.2 | Aminotransferase (CE07462) |
| F35B12.6 | 3.2 | Serine protease inhibitor (CE05805) |
| C14B9.1 | 3.2 | Alpha-B-crystallin (CE00072) |
| T22G5.2 | 3.0 | Fatty-acid binding protein (CE13984) |
| T04C10.4 | 2.8 | Transcription factor ATF4 like (CE06355) |
| T20B3.2 | 2.8 | Troponin (CE20087) |
| F07A5.7 | 2.6 | Paramyosin (CE09197) |
| C37E2.1 | 2.6 | Isocitrate dehydrogenase (CE08620) |
| B0272.5A | 2.6 | Endothelial actin-binding protein repeats (CE07710) |
| C09D4.3 | 2.6 | SER-THR protein kinase (CE27672) |
| K10G9.1 | 2.6 | Sodium-phosphate transporter protein (CE01603) |
| F52B10.1 | 2.6 | Myosin (CE04618) |
| K10C2.1 | 2.6 | Serine carboxypeptidase (CE04747) |
| C02C2.3 | 2.6 | Acetylcholine receptor (CE00029) |
| C11E4.1 | 2.5 | Glutathione peroxidase (CE08101) |
| F54F11.2 | 2.3 | Zinc-binding metalloprotease (CE11095) |
| Y49A3A.2 | 2.3 | ATP synthase alpha and beta subunits (CE22210) |
| M18.2 | 2.3 | Dynein like (CE06194) |
| B0198.1 | 2.3 | Tetraspanin (CE26461) |
| T16G12.1 | 2.1 | Aminopeptidase (CE26001) |
| C05E11.5 | 2.1 | Ammonium transporter (CE27655) |
| B0285.9 | 2.1 | Choline kinase (CE00648) |
| K08B4.3 | 2.1 | Glucuronosyltransferase (CE18032) |
| Y54G11A.5B | 2.1 | Catalase (CE22477) |
| C02B4.2 | 2.0 | Nuclear hormone receptor (CE02111) |
| ZC373.4 | 2.0 | Calcium-calmodulin dependent protein kinase (CE02377) |
| Y57G11C.10 | 2.0 | GDI-1 GDP dissociation inhibitor (CE14944) |
| T02G5.8 | 2.0 | Acetoacetyl-C0A thiolase (CE04860) |
| T19C4.6 | 2.0 | Guanine nucleotide-binding protein (CE06470) |
| C06G8.2 | 2.0 | Oligopeptide transporter (CE05230) |
| C46A5.1 | 2.0 | Protein-tyrosine phosphatase (CE08751) |
| F33C8.3 | 2.0 | Tetraspanin (CE05798) |
| F09F7.4 | 2.0 | Enoyl-CoA hydratase (CE00689) |
