# Supplementary material for: Impact of Cigarette Smoke Exposure on Innate Immunity: A Caenorhabditis elegans Model
Source: PLoS One. 2009 Aug 31;4(8):e6860. doi: 10.1371/journal.pone.0006860 (PMC2729919; doi:10.1371/journal.pone.0006860)
Supplement: Table S2 — Microarray Smoke vs. Air - Increased Genes. This is the raw microarray data showing all genes with a 2-fold or greater increase between smoke exposure and air controls. (0.04 MB DOC) [file pone.0006860.s002.doc]

**Table S2.** *C. Elegans* genes (n = 19) increased by cigarette smoke exposure (Smoke vs. Air)

| **Worm base ID** | **Fold**  **increase** | **Description and protein ID** |
| --- | --- | --- |
| ZK816.5 | 8.0 | Short-chain alcohol dehydrogenase (CE05118) |
| B0213.7 | 6.5 | Chemoreceptor (CE16778) |
| W03G1.7 | 6.1 | Sphingomyelin phosphodiesterase (CE17285) |
| T05A7.9 | 3.7 | Small histidine-alanine-rich protein precursor (SHARP) (CE04895) |
| C53C11.4 | 3.5 | S. muris microneme antigen (CE04267) |
| F15B9.1 | 3.2 | O.volvulus antigen peptide like (CE09406) |
| ZC64.1 | 2.6 | Transposase (CE05047) |
| F28D1.5 | 2.6 | Arabidopsis pathogenesis-related protein 5 like (CE05745) |
| F01D4.1 | 2.5 | UDP-glucoronosyltransferase (CE09153) |
| F15B9.6 | 2.3 | Phospholipase A2 (CE09411) |
| F54F3.3 | 2.3 | Lipase (CE18732) |
| W06D12.3 | 2.3 | Fatty acid desaturase (CE16551) |
| F22B8.5 | 2.1 | 7TM receptor (CE15883) |
| Y46H3A.3 | 2.1 | Heat shock protein (CE22002) |
| K09F5.5 | 2.1 | Zinc finger protein HRX (CE02822) |
| C05C10.4 | 2.1 | Acid phosphatase (CE17370) |
| T21C9.8 | 2.0 | Transthyretin-like family (CE06477) |
| K04C1.3 | 2.0 | Zinc finger, C2H2 type (CE06087) |
| T08D10.2 | 2.0 | Corticosteroid-binding protein (CE03643) |
