# Supplementary material for: Impact of Cigarette Smoke Exposure on Innate Immunity: A Caenorhabditis elegans Model
Source: PLoS One. 2009 Aug 31;4(8):e6860. doi: 10.1371/journal.pone.0006860 (PMC2729919; doi:10.1371/journal.pone.0006860)
Supplement: Table S3 — Microarray Pseudomonas vs. Control - Decreased Genes. This is the raw microarray data showing all genes with a 2-fold or greater reduction between Psuedomonas exposure and air controls. (0.18 MB DOC) [file pone.0006860.s003.doc]

**Table S3.** *C. Elegans* genes (n = 156) increased by PA infection (PA vs. OP50)

| **Worm base ID** | **Fold**  **increase** | **Description and protein ID** |
| --- | --- | --- |
| F35E8.8 | 59.7 | Glutathione S-transferases (CE15958) |
| ZK666.3 | 29.9 | Lectin C-type domain short and long forms (CE16739) |
| C02F5.8 | 18.4 | Tetraspanin (CE00044) |
| C05A9.1 | 17.1 | P-glycoprotein (CE05221) |
| F15B9.6 | 17.1 | Phospholipase A2 (CE09411) |
| G675493 | 16.0 | Cathepsin B-like cysteine proteinase (AAA98788) |
| C04G6.5 | 16.0 | Salt-stress induced peptide (CE06766) |
| H23L24.5 | 14.9 | Glycohydrolase (CE20978) |
| Y65B4BR.1 | 13.9 | Phospholipase (CE25539) |
| R03D7.6 | 13.0 | Glutathione transferase (CE01613) |
| C54D1.2 | 13.0 | C-type lectin (CE06978) |
| T01D3.6B | 11.3 | EGF-like domain, Fibrinogen beta and gamma chains, C-terminal globular domain (CE18164) |
| F28G4.1 | 9.2 | Cytochrome P450 (CE15919) |
| C01G6.7 | 9.2 | 4-coumarate-coA ligase (CE00869) |
| T25B9.7 | 8.6 | UDP-glucuronosyltransferase (CE06506) |
| W03G1.7 | 8.6 | Sphingomyelin phosphodiesterase (CE17285) |
| Y51A2D.13 | 7.5 | Phospholipase D. Active site motifs (CE19209) |
| C23H3.7 | 7.5 | Trehalase (CE19363) |
| R07B1.3 | 7.5 | Membrane glycoprotein (CE01629) |
| ZK945.1 | 6.5 | Esterase (CE01731) |
| F54H5.4 | 6.5 | Zinc finger (CE01967) |
| F54F3.3 | 6.5 | Lipase (CE18732) |
| C02F5.11 | 6.1 | Tetraspanin (CE25750) |
| C04G6.1 | 6.1 | Ser-Thr protein kinase (CE06762) |
| F35B12.2 | 5.7 | Retinol dehydrogenase like (CE05801) |
| Y48A6B.9 | 5.7 | Zinc-binding dehydrogenases (CE19192) |
| C55A6.7 | 5.7 | Alcohol dehydrogenase (CE09004) |
| F25E2.4 | 5.3 | Cytoplasmic intermediate filament (CE07133) |
| T10B9.2 | 4.9 | Cytochrome P450 (CE01656) |
| M60.2 | 4.9 | Contains similarity to Pfam domain PF09412 (CE04775) |
| T28D6.2 | 4.9 | Tubulin alpha subunit (CE16521) |
| T20B3.1 | 4.9 | Carnitate acyltransferase (CE20086) |
| K09C4.1 | 4.9 | Glucose transporter (CE04729) |
| C01B7.4 | 4.9 | Guanylate kinase (CE06728) |
| B0272.4 | 4.6 | Enoyl-CoA hydratase-isomerase (CE00853) |
| T21E8.1 | 4.6 | P-glycoprotein (CE23998) |
| Y57G11C.24C | 4.6 | Epidermal growth factor receptor kinase substrate (CE14976) |
| M6.1 | 4.3 | Intermediate filament proteim A (CE07382) |
| K08E7.7 | 4.3 | LIN-19 like protein (CE11928) |
| F09A5.1 | 4.3 | Tetracycline resistance like protein (CE03173) |
| ZC328.2 | 4.3 | Zinc finger protein (CE15183) |
| F46B6.8 | 4.3 | Lipase (CE05874) |
| Y32H12A.3 | 4.0 | Dehydrogenase (CE21514) |
| Y40D12A.2 | 4.0 | Serine carboxypeptidase (CE19621) |
| T19D12.1 | 4.0 | A. faecalis PHB depolymerase (CE04976) |
| F52E1.7 | 3.7 | Heat shock protein (CE04635) |
| F11A5.10 | 3.7 | Ligand-gated ion channel (CE24896) |
| F08A8.1 | 3.7 | ACYL-COENZYME A OXIDASE (CE17633) |
| ZK1290.5 | 3.7 | Aldo-keto reductase (CE15543) |
| Y75B8A.4 | 3.7 | ATPases associated with various cellular activities (AAA) (CE23016) |
| C56A3.7 | 3.7 | Caveolin (CE15738) |
| G538372 | 3.7 | Steroid hormone receptor family member CNR8 (AAA96984.1) |
| F55B11.1 | 3.7 | Xanthine dehydrogenase (CE16116) |
| C18C4.3 | 3.7 | UDP-glucuronosyltransferase (CE27363) |
| R08E3.1 | 3.5 | LDL receptor-related protein (CE04822) |
| C45B11.3 | 3.5 | Alcohol dehydrogenase (CE05427) |
| F43H9.2 | 3.5 | Serine palmtoyltransferase (CE07246) |
| Y32F6B.3 | 3.5 | RAS-like GTP-binding protein (CE16612) |
| F33D4.1 | 3.5 | Zinc finger protein-hormone receptor (CE20746) |
| K09C8.1 | 3.5 | Na(+)-H(+) exchanger (CE03477) |
| W06B3.2 | 3.5 | Serine-threonine kinase (CDC2-CDC28 subfamily) (CE27220) |
| F13B10.1 | 3.5 | TIR (Toll/ Interleukin 1 Receptor) domain protein (CE15818) |
| D2045.8 | 3.2 | TNF-alpha induced Protein B12 (CE00608) |
| C16H3.2 | 3.2 | Sugar-binding protein (CE08236) |
| F40F8.7 | 3.2 | Zinc finger, C2H2 type (CE05846) |
| F21G4.1 | 3.2 | Rat prostoglandin transporter like (CE09547) |
| T05A7.5 | 3.2 | Galactoside 3(4)-L-fucosyltransferase (CE04891) |
| F22E10.1 | 3.2 | P-glycoprotein (MDR) (CE03260) |
| H06H21.10 | 3.2 | ATPase (CE27180) |
| D2063.1 | 3.2 | Dehydrogenase (CE20628) |
| F56C11.6 | 3.0 | Carboxylesterases (CE17905) |
| F44E5.4 | 3.0 | Heat shock hsp70 proteins (CE18679) |
| T22G5.2 | 3.0 | Fatty-acid binding protein (CE13984) |
| Y46H3A.3 | 3.0 | Heat shock protein (CE22002) |
| K11E4.2 | 3.0 | Src homology domain 2 (CE06180) |
| g9857634 | 2.8 | Galectin LEC-8 (BAB11964.1) |
| g9857640 | 2.8 | Galectin LEC-11 (BAB11967.1) |
| T12G3. | 2.8 | Drosphila REF (2)P like (CE06438) |
| K11D2.2 | 2.8 | Human PHP32 protein like (CE12120) |
| F21F8.4 | 2.8 | Protease (CE09540) |
| Y39D8C.1 | 2.8 | ABC transporter (CE20234) |
| F43G6.4 | 2.8 | Transcriptional regulatory protein (RPD3) (CE20787) |
| T19D12.2 | 2.8 | A. faecalis PHB depolymerase (CE04977) |
| Y75B12B.6 | 2.8 | Phosphatidylinositol-specific phospholipase C (CE20375) |
| E02H1.7 | 2.8 | Zinc finger, C4 type (two domains) (CE01542) |
| C28G1.3 | 2.8 | GTP-binding protein and M protein |
| Y38C9A.2 | 2.6 | GTP-binding protein |
| T27E4.8 | 2.6 | Heat shock protein HSP16-1 (CE14249) |
| G2190266 | 2.6 | 3-keto-acyl-CoA thiolase (CE18418) |
| T17A3.8 | 2.6 | Protein kinase (CE19585) |
| K03H1.5 | 2.6 | Transmembrane and sushi domain (CE03459) |
| F08G2.1 | 2.6 | Core histones H2A, H2B, H3 and H4 (CE07075) |
| K10B3.2 | 2.6 | TC3A transposable element |
| F44C8.3 | 2.6 | Nuclear hormone receptor (CE17824) |
| F55H12.3 | 2.6 | LDL receptor (CE25008) |
| F20C5.4 | 2.6 | Yeast YCY4 like protein (CE03241) |
| C34H3.2 | 2.6 | C2H2-type zinc finger protein (CE23571) |
| F26E4.12 | 2.5 | Glutathione peroxidase (CE09696) |
| F32G8.6 | 2.5 | GTP cyclohydrolase I (CE05795) |
| F10G8.5 | 2.5 | NCS-2 neuronal calcium sensor protein (CE09340) |
| Y48A6B.7 | 2.5 | Cytidine and deoxycytidylate deaminases zinc-binding region (CE19190) |
| F56F10.1 | 2.5 | Peptidase (CE11274) |
| F40F9.9 | 2.5 | MIP transmembrane protein (CE20771) |
| C39E9.2 | 2.5 | Testis-specific protein TPX-1 like (CE05385) |
| C37E2.5 | 2.5 | Homeobox domain (CE08624) |
| F09C3.1 | 2.5 | Ras GTPase-activating protein like (CE23636) |
| F21D5.3 | 2.5 | Laccase like copper oxidase (CE03246) |
| C47E12.4 | 2.3 | Inorganic pyrophosphatase (CE23601) |
| F53C11.3 | 2.3 | 2,4-dienoyl-CoA reductase (CE10908) |
| ZC416.6 | 2.3 | Protease (CE28160) |
| F11A3.1 | 2.3 | 4-coumarate-CoA ligase (CE05585) |
| Y25C1A.13 | 2.3 | Enoyl-CoA hydratase-isomerase (CE21481) |
| C44B7.9 | 2.3 | Peroxisomal membrane protein (CE02547) |
| g8979463 | 2.3 | Leucine-rich repeat-containing G protein-coupledreceptor AAF82248.1) |
| F21A3.2 | 2.3 | Acid phosphatase (CE09516) |
| g4219015 | 2.3 | Serine-threonine kinase SMA-6 (AAD12261.1) |
| T23H4.3 | 2.3 | zinc metalloprotease (CE25126) |
| F28B12.2 | 2.3 | Transcriptional enhancer factor (TEF-1) (CE27143) |
| C18D11.2 | 2.3 | Acyl CoA binding protein (CE18513) |
| Y75B8A.3 | 2.3 | Carboxylesterases (CE23015) |
| B0041.6 | 2.3 | 6-pyruvoyl tetrahydrobioterin synthase (CE07671) |
| R05G6.10 | 2.3 | Cell Division control protein (CE07423) |
| Y51A2D.4 | 2.3 | Sugar (and other) transporters (CE19201) |
| T10B10.8 | 2.3 | Glycogenin like (CE06406) |
| F22A3.4 | 2.3 | Human homeotic protein PBX2 (CE04440) |
| AV203402 | 2.3 | Xanthine dehydrogenase (CE27966) |
| R08H2.1 | 2.3 | Dehydrogenase (CE12594) |
| F25B3.4 | 2.3 | Serine-threonine protein phosphatase (CE05714) |
| T28H10.3 | 2.1 | Vacuolar processing enzyme like (CE14367) |
| F58F9.7 | 2.1 | acyl-coenzyme A oxidase (CE07304) |
| Y43C5A.2 | 2.1 | Fibrinogen beta and gamma chains, C-terminal globular domain (CE19151) |
| F40F9.2 | 2.1 | N-methyl-D-aspartate receptor associated protein (CE05851) |
| T22H6.2 | 2.1 | 1-pyroline-5-carboxylate synthetase (CE03697) |
| g10047306 | 2.1 | Neural RNA-binding protein MSI-1 (BAB13470.1) |
| T14E8.1 | 2.1 | Protein-tyrosine kinase (CE04956) |
| R10H1.1 | 2.1 | Trypsin inhibitor 9 CE02028) |
| F08B1.1 | 2.1 | locus:vhp-1 protein tyrosine phosphatase (CE01899) |
| F56E3.3 | 2.1 | Kinesin-related protein (CE27176) |
| F23B2.12 | 2.1 | Lysosomal carboxypeptidase (CE09592) |
| W02B12.1 | 2.1 | Phospholipase (CE14408) |
| F33E2.2 | 2.1 | Eukaryotic protein kinase domain (CE23702) |
| C32D5.2 | 2.1 | Activin receptor (CE01842) |
| C12D8.11 | 2.0 | ROP-1 ribonucleoprotein RO autoantigen (CE15613) |
| Y54G11A.13 | 2.0 | Catalase (CE22478) |
| K02D7.1 | 2.0 | Phosphorylase (CE17994) |
| E04F6.3 | 2.0 | Hydratase-dehydrogenase-epimerase (CE01215) |
| Y54G11A.5B | 2.0 | Catalase (CE22477) |
| F34H10.3 | 2.0 | Protein S10 (CE01573) |
| B0302.5 | 2.0 | Tyrosine-protein kinase (CE03870) |
| M05D6.7 | 2.0 | Gamma-butyrobetaine,2-oxoglutarate dioxygenase (CE03505) |
| F54F3.4 | 2.0 | Dehydrogenase (CE19894) |
| F55A12.7 | 2.0 | Clathrin coat assembly protein complex 1 medium chain (CE11129) |
| C53B4.7 | 2.0 | GDP-D-mannose dehydratase 9 CE23608) |
| K08F8.3 | 2.0 | Fucosyltransferase (CE03469) |
| T21C9.8 | 2.0 | Transthyretin-like family (CE06477) |
