# Supplementary material for: Impact of Cigarette Smoke Exposure on Innate Immunity: A Caenorhabditis elegans Model
Source: PLoS One. 2009 Aug 31;4(8):e6860. doi: 10.1371/journal.pone.0006860 (PMC2729919; doi:10.1371/journal.pone.0006860)
Supplement: Table S4 — Microarray Pseudomonas vs. Control - Increased Genes. This is the raw microarray data showing all genes with a 2-fold or greater increase between Psuedomonas exposure and air controls. (0.09 MB DOC) [file pone.0006860.s004.doc]

**Table S4.** *C. Elegans* genes (n = 65) decreased by PA infection (PA14 vs. OP50)

| **Worm base ID** | **Fold**  **reduction** | **Description and protein ID** |
| --- | --- | --- |
| F44G3.2 | 34.3 | Arginine kinase (CE16034 |
| W07B8.5 | 13.9 | Thiol protease (CE1468) |
| Y38F1A.6 | 11.3 | Iron-containing alcohol dehydrogenases (CE24222) |
| F37B4.7 | 11.3 | Folate transporter (CE17796) |
| C52E4.1 | 9.8 | Cathepsin-like cysteine protease (CE08943) |
| C49F5.1 | 6.5 | S-adenosylmethionine synthetase (CE08852) |
| K02D7.3 | 5.3 | Cuticular collagen (CE17996) |
| F32D8.4 | 5.3 | Lactate dehydrogenase (CE23698) |
| T08H10.1 | 4.6 | Aldose reductase (CE17230) |
| F09F7.4 | 3.7 | Enoyl-CoA hydratase (CE00689) |
| B0286.3 | 3.7 | Saicar synthetase-air carboxlyase (CE03863) |
| H12C20.3 | 3.7 | Nuclear hormone receptor (CE18808) |
| F18E3.7 | 3.2 | D-amino acid oxidase (CE07083) |
| B0334.1 | 3.2 | Transthyretin-like family 9 CE02932) |
| T05G5.6 | 3.0 | Enoyl-CoA hydratase (CE00318) |
| T07C12.7 | 3.0 | Transthyretin-like family (CE06389) |
| F38B2.4 | 3.0 | Adenylate kinase (CE02218) |
| C15H9.7 | 3.0 | Kynureninase (CE06835) |
| M88.1 | 3.0 | UDP-glucuronosyltransferase (CE01036) |
| M02F4.8 | 2.8 | MIP family protein (CE04769) |
| M163.3 | 2.8 | Histone H1 (CE12450) |
| T05D4.1 | 2.8 | Fructose-bisphosphate aldolase class-I (CE16341) |
| K08C7.3 | 2.8 | Laminin (CE25049) |
| F14D12.2 | 2.8 | LIM domain containing protein (CE04392) |
| F54D5.12 | 2.6 | Yeast hyothetical YET1 like protein (CE26814) |
| W05G11.6 | 2.6 | Phosphoenolpyruvate carboxykinase (CE19610) |
| C04F6.3 | 2.6 | Glycosyl hydrolase (family 18) (CE03923) |
| Y53F4B.30 | 2.6 | Glutathione-S-transferase (CE22417) |
| C11E4.1 | 2.6 | Glutathione peroxidase (CE08101) |
| F46G10.6 | 2.6 | Helix-loop-helix DNA-binding domain (CE05882) |
| T01B11.2 | 2.6 | Aminotransferase (CE12894) |
| G2407320 | 2.5 | Histone H1.1 (AAB70665.1) |
| K09H11.7 | 2.5 | Nitrophenylphosphatase (CE12060) |
| F13D12.2 | 2.5 | L-lactate dehydrogenase (CE02181) |
| C25B8.4 | 2.5 | Asialoglycoprotein receptor (CE27087) |
| F01F1.6 | 2.5 | Aldehyde dehydrogenase (AAC46640.1) |
| F28A10.6 | 2.5 | Acyl-coA dehydrogenase 9 CE19411) |
| C55A6.1 | 2.5 | Zinc finger, C3HC4 type (RING finger) (CE08998) |
| C10C5.5 | 2.5 | Aminoacylase-1 (CE03013) |
| F32H2.6 | 2.5 | Fatty acid synthase (N-terminus) (CE09881) |
| T02G5.8 | 2.3 | Acetoacetyl-C0A thiolase 9 CE04860) |
| K10C2.3 | 2.3 | Aspartyl protease (CE04749) |
| T13F2.8 | 2.3 | Caveolin (CE13633) |
| F58B3.9 | 2.3 | Transthyretin-like family (CE11362) |
| F28F8.2 | 2.3 | Long-chain-fatty-acid-CoA ligase (CE09756) |
| F18E2.1 | 2.3 | Acid phophatase like (CE05662) |
| K07E3.3 | 2.3 | Methylenetetrahydrofolate dehydrogenase (CE25047) |
| C14H10.1 | 2.3 | Yeast YIL023C-like protein (CE23542) |
| K06A4.5 | 2.3 | 3-hydroxyanthranilate 3,4-dioxygenase (CE06109) |
| Y69E1A.5 | 2.3 | Phosphatidylethanolamine-binding protein (CE22812) |
| C05C8.3 | 2.3 | FKBP-binding protein (CE07921) |
| T04C10.4 | 2.1 | Transcription factor ATF4 like (CE06355) |
| C44F1.3 | 2.1 | Galactoside binding lectin 9 CE02163) |
| F42E11.4 | 2.1 | Troponin I (CE03311) |
| C30F12.7 | 2.1 | Isocitrate dehydrogenase (CE16887) |
| F55D10.1 | 2.1 | Alpha-mannosidase (CE28456) |
| T22E5.5 | 2.0 | Troponin T-like protein (CE04994) |
| R03D7.1 | 2.0 | 5-methyltetrahydrofolate-homocysteine methyltransferase (CE01609) |
| T11F9.3 | 2.0 | Zinc metalloprotease (CE06415) |
| C55F2.1 | 2.0 | Transferase (CE26363) |
| G1203791 | 2.0 | Steroid hormone receptor family member CNR14 (CE03323) |
| K10H10.2 | 2.0 | Beta-synthase (CE16252) |
| F13C5.2 | 2.0 | Bromodomain-containing protein (CE19384) |
